# A Low-Cost IoT Sensor and Preliminary Machine-Learning Feasibility Study for Monitoring In-Cabin Air Quality: A Pilot Case from Almaty

**DOI:** 10.3390/s25144521

**Published:** 2025-07-21

**Authors:** Nurdaulet Tasmurzayev, Bibars Amangeldy, Gaukhar Smagulova, Zhanel Baigarayeva, Aigerim Imash

**Affiliations:** 1 Institute of Combustion Problems, Almaty 050012, Kazakhstan; tasmurzayev.n@gmail.com (N.T.); iimash.aigerim@gmail.com (A.I.); 2 LLP “DigitAlem”, Almaty 050042, Kazakhstan; 3 Faculty of Information Technologies, Al-Farabi Kazakh National University, Almaty 050040, Kazakhstan; zhanel.baigarayeva@gmail.com; 4 Department of General Physics, Intistute of Energy and Mechanical Engineering Named after A. Burkitbayev, Satbayev University, Almaty 050013, Kazakhstan; 5 Department of Chemistry, Abai Kazakh National Pedagogical University, Almaty 050010, Kazakhstan; 6 LLP “Kazakhstan R&D Solutions”, Almaty 050056, Kazakhstan

**Keywords:** indoor air quality (IAQ), public transport, air pollution monitoring, machine learning, passenger density, bus, metro, trolleybus

## Abstract

The air quality within urban public transport is a critical determinant of passenger health. In the crowded and poorly ventilated cabins of Almaty’s metro, buses, and trolleybuses, concentrations of CO_2_ and PM_2.5_ often accumulate, elevating the risk of respiratory and cardiovascular diseases. This study investigates the air quality along three of the city’s busiest transport corridors, analyzing how the concentrations of CO_2_, PM_2.5_, and PM_10_, as well as the temperature and relative humidity, fluctuate with the passenger density and time of day. Continuous measurements were collected using the Tynys mobile IoT device, which was bench-calibrated against a commercial reference sensor. Several machine learning models (logistic regression, decision tree, XGBoost, and random forest) were trained on synchronized environmental and occupancy data, with the XGBoost model achieving the highest predictive accuracy at 91.25%. Our analysis confirms that passenger occupancy is the primary driver of in-cabin pollution and that these machine learning models effectively capture the nonlinear relationships among environmental variables. Since the surveyed routes serve Almaty’s most densely populated districts, improving the ventilation on these lines is of immediate importance to public health. Furthermore, the high-temporal-resolution data revealed short-term pollution spikes that correspond with peak ridership, advancing the current understanding of exposure risks in transit. These findings highlight the urgent need to combine real-time monitoring with ventilation upgrades. They also demonstrate the practical value of using low-cost IoT technologies and data-driven analytics to safeguard public health in urban mobility systems.

## 1. Introduction

Serious air quality issues are a persistent challenge in public transport across many tropical and subtropical nations. However, research into this problem is disproportionately concentrated in tropical Asia (which accounts for 78.2% of studies), which has left significant knowledge gaps in regions like Sub-Saharan Africa, South America, and Oceania [1]. This issue is often exacerbated in developing countries, which typically rely on aging vehicle fleets with poor ventilation. Such conditions lead to high in-cabin concentrations of pollutants, including PM_2.5_, PM_10_, CO, and VOCs [1,2]. Long-term monitoring in Jinan City shows that the annual mean PM_2.5_ dropped by about 60% between 2014 and 2021, while the overall AQI decreased by 27%, which demonstrates that sustained policy action can reverse urban-pollution trends [3]. When combined with overcrowding and inadequate infrastructure, poor air quality in public transport significantly elevates public health risks [4]. This situation is mirrored in Kazakhstan, where ambient air pollution is a severe national issue. In 2021, for instance, a staggering 2.1 million tons out of the nation’s 2.4 million tons of emissions originated from its Central region. In major cities, the PM_2.5_ levels have reached alarming heights, exceeding WHO standards by 19.8 times in Astana and 20.5 times in Karaganda. Research has established a strong correlation in the country between air pollution and adverse health outcomes, including respiratory diseases (r = 0.97) and mortality (PM_2.5_: r = 0.97; CO_2_: r = 0.75) [5]. Within this context, Almaty’s air quality is a critical environmental concern, with a demonstrable negative impact on public health [6]. According to the World Health Organization (WHO), the PM_2.5_ concentrations in the city can exceed permissible limits by as much as 17 times during the winter. This severe pollution is directly linked to higher rates of illness and increased mortality, particularly among vulnerable populations [7].

Exposure to poor air quality on public transport is associated with a wide range of health problems. These include acute respiratory infections, cardiovascular disease, and stress disorders, and these conditions also facilitate the spread of airborne viruses like influenza and COVID-19 [1,4]. For daily commuters, continuous exposure can lead to increased work absenteeism and a growing societal disease burden. High concentrations of pollutants such as PM_2.5_, PM_10_, CO_2_, CO, VOCs, and allergens carry long-term consequences, from allergic reactions and respiratory symptoms to carcinogenic risks [8]. Key pollutants like VOCs and toxic microelements often exceed safe thresholds, especially in developing nations where vehicle ventilation systems are insufficient for proper air filtration [1]. Beyond physical health, poor air quality also degrades the comfort and safety of passengers and drivers alike, diminishing their overall well-being and potentially increasing the risk of traffic accidents [9]. This problem is compounded by overcrowding, which raises CO_2_ levels, and by outdated HVAC systems in older vehicles that fail to meet modern ventilation standards. Addressing these deficits requires a shift toward modern HVAC technology, high-efficiency filters, and innovative vehicle solutions like electric and hybrid models to significantly improve the air quality on public transit and mitigate health risks [1].

Rapid urbanization drives both passenger density and transport activity, which in turn degrades the air quality inside public transport [9,10]. As urban populations grow, the demand for transit services intensifies, which leads to more frequent trips and higher pollution from vehicle emissions [10,11,12]. This is particularly true where transport infrastructure is still developing and ventilation systems cannot cope, which results in poor air quality inside vehicles and at transport hubs. Studies confirm that high passenger density is a key factor that influences the microclimate of enclosed transit spaces [10,13]. For example, an analysis of the Beijing subway revealed that the passenger density varies dramatically with time. On weekdays, morning (06:00–09:00) and evening (17:00–20:00) peaks cause passenger loads to exceed the system’s designed capacity, compromising fresh air intake and air quality. In contrast, the passenger flow on weekends is significantly lower—just 57.2% of the weekday volume—allowing for healthier conditions [13]. These fluctuations in occupancy directly impact passengers’ comfort and exposure to pollutants, underscoring the need for time-sensitive environmental monitoring. While many studies have investigated the indoor air quality (IAQ) in public transport, a significant research gap remains in understanding the minute-by-minute effects of passenger density on pollutant accumulation—particularly in the unique context of a rapidly growing Central Asian megacity like Almaty. Here, a combination of overcrowded vehicles, aging ventilation systems, and a single, heavily used metro line creates distinct public health challenges.

The primary contribution of this work is the design and development of a low-cost, end-to-end monitoring system to fill this gap. Our interdisciplinary approach integrates custom hardware engineering (the Tynys mobile IoT sensor (Laboratory in Institute of Combustion Problems, Almaty, Kazakhstan)), a cloud infrastructure for real-time data transmission, and data science for advanced analysis. For our monitoring platform, we selected the Winsen ZPHS01B multisensor module (Zhengzhou Winsen Electronics Technology Co., Ltd., Zhengzhou, China). This device was chosen not only for its comprehensive suite of sensors but also because its performance has been independently assessed in the scientific literature. A recent study by the authors of [14] evaluated the ZPHS01B against regulated reference stations, demonstrating that its PM_2.5_ and O_3_ sensors show a strong correlation (R^2^ ≈ 0.8) after calibration, which makes it a reliable component for air quality monitoring deployments. Our research leverages this validated hardware as the core of a novel, end-to-end system designed for the challenges of monitoring in-transit environments. A key aspect of the originality of our approach is demonstrating that such an integrated system is not only technically feasible but also highly effective at capturing high-resolution environmental data in dynamic transport environments. This is the first study to quantitatively assess the real-time, minute-by-minute impact of passenger density on the in-cabin air quality across buses, trolleybuses, and the Almaty metro. By training multiple machine learning algorithms on the data we collected, we validated that an XGBoost model could classify pollution levels with 91.25% accuracy. This result provides a critical and unique dataset for Almaty and serves as a powerful proof-of-concept for our methodology. Therefore, we present this system not merely as a localized study but as a scalable and affordable blueprint that other cities facing similar transport-related air quality issues can adopt and adapt.

## 2. Literature Review

The air quality within urban public transport is a significant environmental concern, primarily due to emissions of particulate matter (PM_2.5_, PM_10_), gases (CO_2_, CO), and volatile organic compounds (VOCs) that directly impact passenger health [15,16]. Air quality profiles vary dramatically between different modes of transport [17]. In Barcelona’s metro, for instance, while the concentration of the smallest nanoparticles is lowest, the dominant particles are larger (90 nm modal size) compared to those found on buses, on trams, or on the street. The concentrations of PM_2.5_ and black carbon in the metro are typically lower than on buses but higher than on trams [17,18]. In Athens, the PM_2.5_ levels on metro platforms can be from 3 to 10 times higher than the ambient street levels, particularly in deep, crowded stations. These particles are often characterized by a high iron content from the wear of wheels and brake pads. In contrast, diesel buses tend to have the highest nanoparticle concentrations, exceeding 5.0 × 10^4^ particles/cm^3^, along with elevated levels of antimony, copper, and xylene from fuel use [17,18]. Trams generally offer a cleaner environment, with lower concentrations of PM_2.5_ and black carbon than other transit modes. A common issue across all vehicle types is the frequent opening of doors, which allows external pollutants to infiltrate and degrade the in-cabin air quality [16,18].

Passenger density is another critical factor that influences indoor air quality, increasing the accumulation of pollutants and the risk of cross-contamination. Passengers themselves act as sources of bioeffluents, including CO_2_, microbes, and aerosols, while their movement also contributes to the resuspension of settled dust and particles [16,18]. CO_2_ levels, in particular, will show a linear relationship with the number of occupants, often exceeding 1200 ppm in overcrowded buses and trains [18]. In such conditions, ventilation systems frequently fail to mitigate the buildup of pollutants, heightening the risk of airborne disease transmission [19]. Interestingly, one study noted that the PM_2.5_ concentrations could decrease with more passengers—theoretically due to inhalation—although no definitive conclusion on this relationship was reached [18].

To understand and mitigate these issues, various technological solutions for monitoring air quality have been deployed [19]. Real-time instruments like the DustTrak II and Air Scan devices are used on buses to capture minute-by-minute fluctuations in particulate matter that are caused by road-dust resuspension and onboard activity. These sensors reveal clear correlations between the in-cabin and outdoor pollution, allowing for the assessment of air-cleaning technologies [20]. Mobile IoT platforms, such as the Libelium Waspmote (Libelium Comunicaciones Distribuidas S.L., Zaragoza, Spain), have become an effective way to obtain detailed spatiotemporal data. By transmitting data via 4G to cloud servers, these wireless sensor networks can generate dynamic air quality maps and identify pollution hotspots along transit routes [21]. These low-cost mobile sensors complement fixed monitoring stations, increasing the density and resolution of data for advanced modeling and real-time air quality control. The integration of mobile and stationary data through geostatistical methods and machine learning allows authorities to implement targeted interventions based on current conditions [22].

Although reliable monitoring is critical, improving air quality ultimately depends on effective ventilation and filtration strategies. A leading option is the installation of air-cleaning systems that combine high-efficiency particulate air (HEPA) filters with ultraviolet (UV-LED) light. HEPA filters can lower PM_2.5_ concentrations by 34–60% and PM_10_ concentrations by 25–61%, while UV-LED technology inactivates airborne bacteria and viruses, reducing the risk of infection. These devices are often mounted centrally within a cabin to optimize the airflow and ensure uniform purification [20]. In metro systems, the installation of platform screen doors is an effective infrastructural change that limits the transfer of contaminants from tunnels into platform areas, and thus significantly reduces concentrations of particulate and microbial pollutants when combined with advanced HVAC systems [23]. Beyond HEPA, other technologies like photocatalytic oxidation (PCO), ionization, and biofiltration are under active investigation. While these methods have proven effective in removing gaseous pollutants and promoting particle deposition in stationary settings, their adaptation to the dynamic conditions of buses and trains is still ongoing [24].

The rapid advancement of the internet of things (IoT) has transformed air quality management in public transport. The integration of sensor networks with wireless communication protocols and cloud analytics now enables the continuous, high-resolution, real-time monitoring of ambient conditions, paving the way for smarter and more responsive public health interventions [25].

Vehicle-mounted IoT sensors and edge networks are emerging as a powerful approach for creating efficient, highly localized air-quality monitoring systems. The use of these networks typically involves installing sensors on public and private vehicles to continuously collect data on pollutants like PM_2.5_, PM_10_, NO_2_, O_3_, SO_2_, and CO [26]. The COCAL project in Trieste, for example, mounts optical sensors on volunteer vehicles to track particulate matter, using integrated probes to obtain environmental context and correcting the daily bias against a fixed reference station [27]. Another innovative prototype demonstrates an energy-saving approach, using an Arduino-based system with a suite of sensors (Plantower PMS5003, MQ-series (Beijing Plantower Technology Co., Ltd., Beijing, China)) that transmits data only when WHO thresholds are exceeded [26].

The true power of this approach lies in its ability to turn raw measurements into actionable intelligence. On-site processing via edge computing enables the immediate detection of pollution peaks, while the data from the entire vehicle sensor network can be aggregated to build real-time pollution distribution maps for a city [27]. To create an even richer picture, advanced data-fusion techniques integrate these mobile data with records from fixed monitoring stations, meteorological information, and traffic-flow data. Methods such as kriging—sometimes enhanced with external-drift techniques—and adaptive hybrid models are used to interpolate pollutant levels at unsampled locations. The result is high-resolution, dynamic air-quality maps that capture both short-term fluctuations and long-term trends [28,29].

This ability to generate detailed maps allows authorities to move from passive monitoring to targeted action, for instance by installing real-time public displays in areas with a consistently high population density and pollution levels [16]. Once such pollution hotspots are identified, a key solution lies in enhancing the efficiency of ventilation [30]. A variety of strategies can be employed, from increasing local exhaust to remove pollutants with existing systems to implementing mixed or personal ventilation systems to reduce occupant exposure and cross-contamination [18,19].

In specific transit environments, these strategies become more tailored. For metro systems, ventilation can be dynamically controlled using feedback from PM_10_ sensors to improve the air quality on platforms [18]. For vehicles, existing HVAC systems can be optimized with variable frequency drives to boost their energy efficiency while maintaining comfortable and safe in-cabin conditions [31]. To design and validate these solutions before implementation, engineers increasingly rely on advanced tools like computational fluid dynamics (CFD) to analyze air distribution patterns and optimize the performance of ventilation systems [19]. A recent CFD study that combined one extractor fan with two plant-based organic air cleaners lowered classroom CO_2_ levels to 613 ppm while keeping the temperatures within the ASHRAE 55 comfort range, showing that low-cost retrofits can improve IAQ without sacrificing occupant comfort [32]. Computational fluid dynamics (CFD) has become an indispensable tool for optimizing the microclimate within vehicle cabins. By providing a three-dimensional analysis of transient airflow, CFD models are especially crucial for designing HVAC systems in confined spaces where passenger seating and spatial configuration create non-uniform air mixing. Detailed models, such as unsteady Reynolds-averaged Navier–Stokes (URANS) with SST k–ω turbulence schemes, can capture the complex interactions among the HVAC supply flow, passenger-induced thermal plumes, and pollutant dispersion. Studies demonstrate that aerosol trajectories and concentrations depend heavily on an infected occupant’s position and the selected ventilation mode, which makes CFD essential for assessing both safety protocols and energy efficiency [31]. The ability of these models to resolve local velocity gradients helps identify stagnation zones and recirculation pockets that are critical for effective pollutant removal [33]. Incorporating passenger thermal loads further refines these predictions, offering a more realistic simulation of operating conditions in densely occupied cabins [34].

However, despite the power of such advanced modeling tools, significant gaps persist in the understanding and control of air quality in public transport. Key uncertainties remain regarding the quantitative impact of passenger density on pollutant transmission, and a consistent link between the occupancy and PM concentration has yet to be established. The airborne spread of viruses in transit environments is also under-researched, and there is still no scientific consensus on which characteristics of particulate matter are most closely linked to specific health outcomes. Furthermore, existing monitoring systems often lack the capability of capturing dynamic fluctuations in pollution in real time, while the integration of data from various sensors presents significant methodological challenges. A clear lack of comparative studies on air flow and exchange rates across different transport modes further complicates the development of universal standards. These gaps underscore a pressing need for further research, particularly research that integrates real-world passenger flow data with robust environmental monitoring.

Addressing these issues is critical in rapidly growing Central Asian megacities like Almaty, where overcrowded vehicles and outdated ventilation systems create unique exposure risks. To fill this research gap, we developed and deployed an occupancy-aware monitoring system. This system combines our custom, low-cost internet-of-things (IoT) sensor, the Tynys, with a suite of machine-learning algorithms, including XGBoost, random forest, and others. The Tynys sensor streams geo-referenced measurements of CO_2_, PM_2.5_, and PM_10_ concentrations, as well as the temperature and humidity, every minute. To our knowledge, this is the first study to quantify how real-time passenger density influences the in-cabin air quality across Almaty’s buses, trolleybuses, and its single metro line. Our best-performing models achieved a classification accuracy above 91% and detected hazardous peak-hour CO_2_ spikes exceeding 2800 ppm. The resulting dataset provides a critical foundation for implementing dynamic ventilation control and optimizing transit schedules on Almaty’s most congested routes, serving as a scalable blueprint for other cities that are facing similar transport-related air-quality challenges.

## 3. System Description

This section details the architecture and operation of the real-time air quality monitoring system developed for this study. At its core is the Tynys, a custom-engineered mobile internet of things (IoT) device that integrates a suite of gas, particulate matter, and environmental sensors with an embedded processing unit. The platform was designed specifically for deployment in dynamic, enclosed environments such as buses, metro trains, and trolleybuses, allowing for fully autonomous data acquisition with minimal user intervention.

To enable real-time analysis, the system continuously samples, filters, and transmits sensor data wirelessly. On the device, measurements are processed using a lightweight moving average filter before being immediately transmitted via Wi-Fi to a centralized server. These data are then stored in a structured SQL database and rendered on a cloud-connected dashboard, which provides operators with a live view of indoor air quality conditions. To validate its performance, the Tynys device was rigorously benchmarked against a commercial, reference-grade air quality monitor under identical operating conditions.

**Figure 1:**
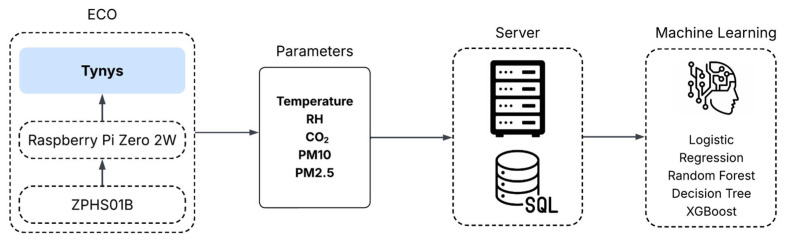
System architecture.

The overall system architecture is structured into four hierarchical layers, as illustrated in Figure 1: (1) the mobile sensing and preprocessing unit (Tynys), (2) an embedded communication interface, (3) a cloud-based data infrastructure, and (4) a machine learning and analytics layer. This modular, layered design facilitates scalable deployment and enables end-to-end automation of the entire data pipeline, from acquisition to analysis.

The foundational layer of the system is the mobile sensing and preprocessing unit, which is embodied by the Tynys IoT device. This component is responsible for localized data acquisition and initial signal filtering. Its structural design, sensor suite, and onboard processing routines are discussed in detail in Section 3.1. The second layer manages the bidirectional wireless communication between the Tynys device and the cloud backend. The processed sensor data are timestamped, formatted as CSV, and transmitted over Wi-Fi using the lightweight MQTT protocol. To ensure the device’s robust performance in mobile environments with intermittent connectivity, this layer incorporates a buffer-based fallback mechanism. This feature temporarily stores data during transmission delays and automatically uploads the buffered data once connectivity is restored, thereby preserving temporal continuity and data completeness. At the third layer, the cloud infrastructure ingests all transmitted data into a structured SQL database hosted on a secure server. This backend enables high-frequency data retrieval, persistent storage, and reliable access for any downstream processing. A cloud-connected web dashboard provides authorized users with interactive visualizations of both historical and real-time air quality metrics, complete with the capability to filter the data based on the location, timestamp, or transport type. The architecture is designed for scalability and secure multi-user access, which makes it suitable for future integration into smart city or transit management platforms. The fourth and final layer consists of a machine learning pipeline designed for pollution level classification and anomaly detection. The system supports multiple supervised learning algorithms—including logistic regression, decision tree, random forest, and XGBoost—which are trained on historical IAQ data using features such as CO_2_, PM_2.5_, PM_10_, temperature, and relative humidity measurements. This analytics module generates real-time predictions that can trigger alerts or inform long-term policy decisions. Integrated directly with the cloud infrastructure, the analytics engine provides intelligent decision support for transit operators, public health authorities, and urban planners.

### 3.1. Tynys IoT Device

The Tynys device is a portable IAQ monitor that is purpose-built for enclosed buses, trolleybuses, and metro cars, and enables real-time environmental assessment. We selected the ZPHS01B multisensory (Zhengzhou Winsen Electronics Technology Co., Ltd., Zhengzhou, China) as the base of the Tynys device. The selection of the ZPHS01B multisensor module was predicated on its suitability for low-cost, high-density deployments and, critically, its prior validation in peer-reviewed research. Reference [14] provides a detailed case study of the ZPHS01B’s performance in a real-world urban setting, comparing it directly with a regulated air quality monitoring station (AQMS). Their findings indicate that the PM_2.5_ and O_3_ sensors are the most reliable components of the module, capable of achieving a coefficient of determination (R^2^) of approximately 0.8 through a linear regression calibration. The authors also noted limitations of the other integrated sensors, such as the NO_2_ sensor’s tendency for saturation and the CO sensor’s low activation levels in non-extreme conditions. These published findings align with our own observations and provide a strong, evidence-based justification for utilizing the ZPHS01B module as a robust tool for tracking key pollutants like particulate matter, while acknowledging the known characteristics of its other sensors.

**Figure 2:**
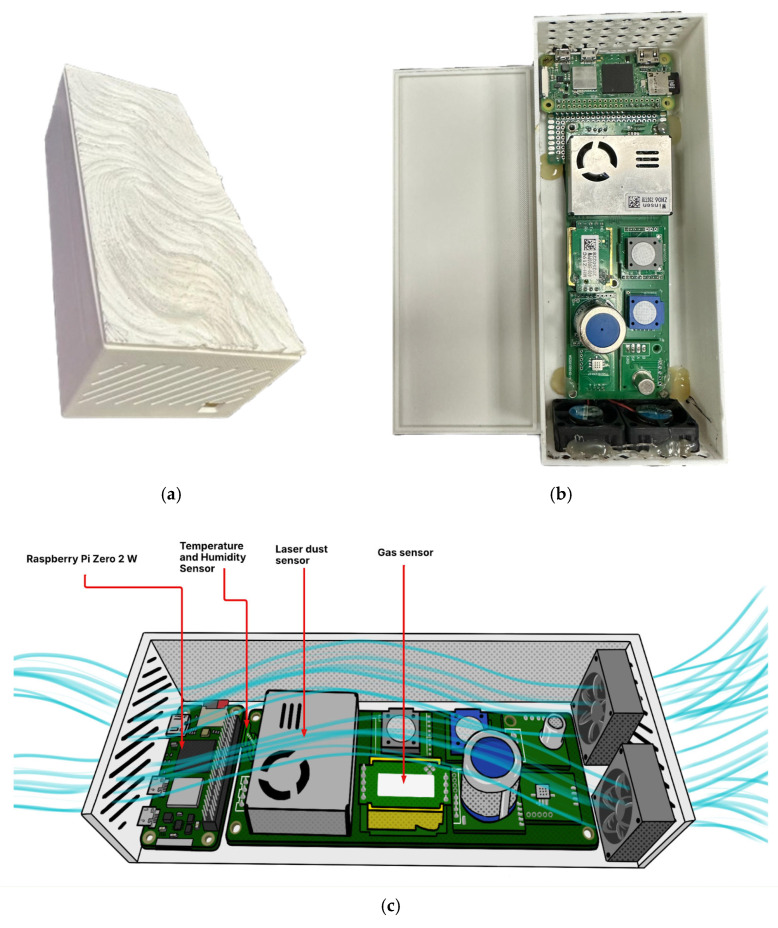
External enclosure of the Tynys device for real-time air-quality monitoring: (**a**) 3D-printed housing with ventilation slots and power connector; (**b**) textured front panel; (**c**) internal layout schematic—Raspberry Pi Zero 2 W, temperature-humidity sensor, laser dust sensor, gas sensor, and fans.

The Tynys device, as shown in Figure 2, is housed in a custom-designed, ventilated enclosure that is tailored for mobile deployment. The primary processing unit is a Raspberry Pi Zero 2 W microcontroller (Raspberry Pi Ltd., Inazawa, Japan), which interfaces with an array of embedded sensors to collect environmental data. The microcontroller executes all acquisition routines, filters sensor signals in real time, and manages wireless data transmission. The sensor readings are sampled at a fixed interval of 60 s and preprocessed using a five-point moving average algorithm to attenuate noise and outlier effects. The filtered data are timestamped, formatted in CSV structure, and transmitted via Wi-Fi using the MQTT protocol.

The sensing module integrates a suite of precision sensors to monitor key indoor air quality parameters. The carbon dioxide (CO_2_) concentration is measured using the MH-Z19C non-dispersive infrared (NDIR) sensor (Zhengzhou Winsen Electronics Technology Co., Ltd., Zhengzhou, China), which covers a range of 0–5000 ppm with an accuracy of ±50 ppm. The particulate matter levels, specifically those of PM_2.5_ and PM_10_, are measured using the ZH06 laser-based optical sensor, which offers a minimum detection limit of 0.3 μm. The temperature and relative humidity are recorded using the ZS05 digital sensor (Zhengzhou Winsen Electronics Technology Co., Ltd., Zhengzhou, China), with measurement accuracies of ±0.3 °C and ±3% RH, respectively.

### 3.2. Validation and Calibration of Tynys IoT Device

To evaluate the data consistency of the Tynys device, a two-phase procedure was conducted that involved both pre-deployment calibration and real-world validation. The calibration was performed over a one-hour session under controlled indoor conditions, during which the Tynys device was operated alongside the Qingping Air Quality Monitor Gen 2. This step allowed the alignment of the baseline sensor outputs and the minimization of the initial offsets through synchronized measurements across key environmental parameters.

The Qingping Air Monitor, which was used in this study as a comparative device for evaluating the Tynys platform, has previously undergone independent testing to assess its performance. In a two-month field evaluation conducted by the South Coast Air Quality Management District (SCAQMD), three Qingping units were co-located with certified FEM-grade instruments (GRIMM EDM180 and Teledyne T640) at an official ambient monitoring site. Although the results of this evaluation are considered preliminary, the Qingping devices demonstrated strong to very strong correlations in PM_2.5_ mass concentrations compared to the reference analyzers (R^2^ = 0.89–0.95 for 1-h means). The devices also achieved 100% data recovery, low intra-model variability (~0.43 µg/m^3^), and mean absolute errors ranging from 0.9 to 2.0 µg/m^3^ depending on the averaging interval [35].

After calibration, validation was carried out in a mobile deployment environment. Both devices were installed side by side within a passenger cabin to ensure simultaneous exposure to identical conditions, and measurements were recorded at regular intervals in keeping with the full-scale protocol.

**Figure 3:**
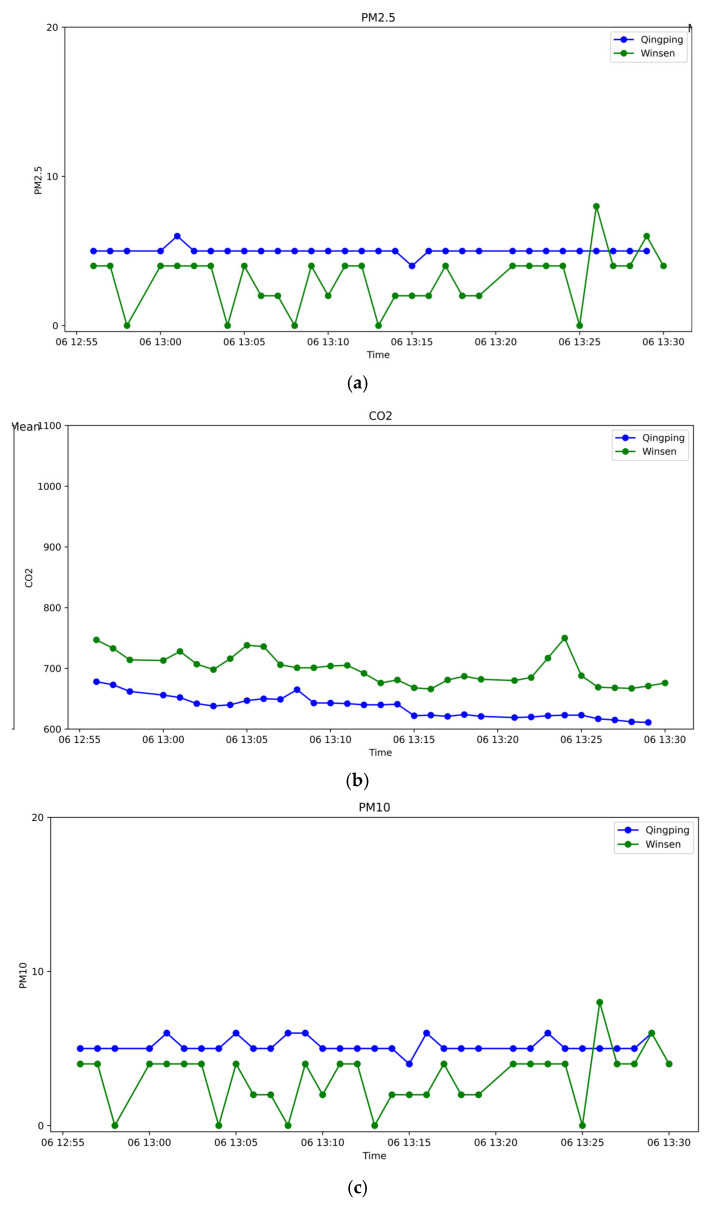

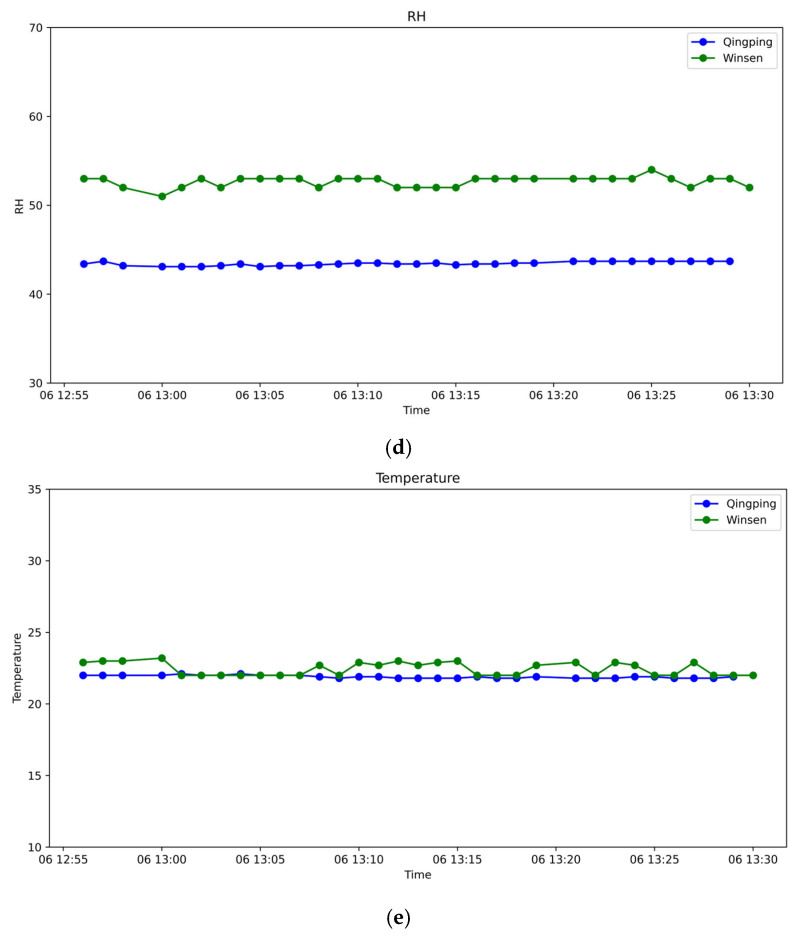
Time-series comparison of (**a**) PM_2.5_; (**b**) CO_2_; (**c**) PM_10_; (**d**) relative humidity; (**e**) temperature recorded by the Qingping and Tynys devices during validation.

Figure 3 shows time-series comparisons of PM_2.5_, PM_10_, CO_2_, temperature, and relative humidity measurements. Across all parameters, the Tynys device tracked ambient fluctuations with good fidelity. The PM readings mirrored those of the Qingping monitor, the CO_2_ trends overlapped closely, and the temperature and humidity remained well aligned.

## 4. Methodology

### 4.1. Experiment Description

All experiments were conducted in April 2025 across three types of public transportation in Almaty: buses, trolleybuses, and metro trains. These three modes, the city’s primary means of mobility, were chosen to explore how passenger density shapes IAQ under routine operating conditions. The rationale behind this selection was to capture diverse occupancy dynamics and infrastructural conditions across metro lines. By including stations from the northern (M1), southern-western (M2), and central (M3) segments, this study ensured spatial representativeness and enabled the evaluation of how indoor air quality (IAQ) varies across different microenvironments and urban settings. This comprehensive spatial sampling supports a robust comparison of pollutant trends and validates the real-world performance of the Tynys IoT device under varying operational conditions.

To ensure comprehensive spatial coverage, three representative routes were selected for each transport mode. The selection criteria included the route length, population density, and traffic intensity.

**Figure 4:**
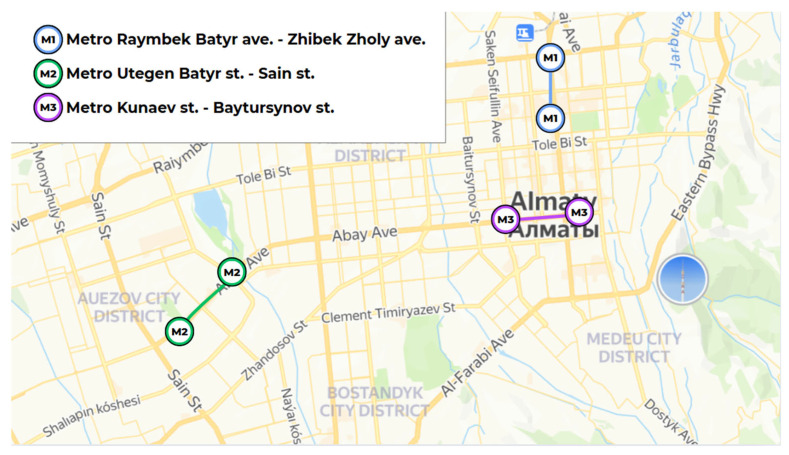
Selected metro segments in Almaty used for IAQ monitoring: M1 (Rayymbek Batyr–Zhibek Zholy), M2 (Utegen Batyr–Sain), and M3 (Kunaev–Baytursynov).

Figure 4 illustrates the spatial distribution of metro segments selected for air quality monitoring in Almaty, Kazakhstan. The marked stations—Raiymbek Batyr, Uttegen Batyr, Kunaev, Zhibek Zholy, Sain, and Baytursynov—represent key intersections across three metro lines (M1, M2, M3) and were strategically chosen based on passenger traffic density, geographic coverage, and urban zoning characteristics.

These stations serve as major transit nodes during peak commuting hours (08:00–09:00), particularly Raiymbek Batyr and Zhibek Zholy, which lie in the historical and commercial heart of the city and handle substantial footfall due to their proximity to business centers and intermodal links. It is important to note that Almaty Metro operates a single unbranched line (Line 1), spanning approximately 13.4 km, with 11 stations. The absence of alternate lines or branches concentrates commuter flow into a single axis, exacerbating occupancy and ventilation challenges during peak hours [36]. Abay and Auezov were included to represent high-occupancy corridors that serve educational and administrative zones, while Moskva and Saryarqa were selected for their role in connecting residential areas to the urban core, where crowding and limited ventilation are recurrent issues.

**Figure 5:**
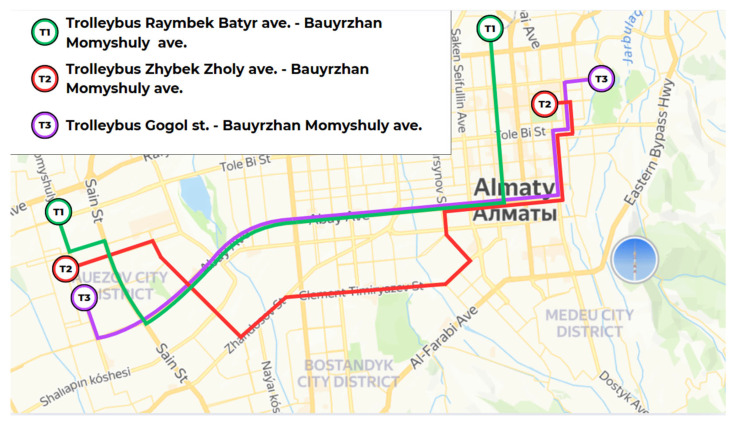
Trolleybus routes T1–T3 selected for IAQ measurement, connecting residential, commercial, and peripheral areas of Almaty.

Figure 5 illustrates the spatial distribution of metro segments selected for air quality monitoring in Almaty, Kazakhstan. The marked stations—Raiymbek Batyr, Uttegen Batyr, Kunaev, Zhibek Zholy, Sain, and Baytursynov—represent key intersections across three metro lines (M1, M2, M3) and were strategically chosen based on passenger traffic density, geographic coverage, and urban zoning characteristics.

Figure 5 presents the trolleybus routes selected for air quality measurements in Almaty, spanning major longitudinal corridors that connect the eastern and western extents of the city. The selected lines—T1 (Rayimbek Batyr Ave.–Bauyrzhan Momyshuly Ave.), T2 (Zhybek Zholy Ave.–Bauyrzhan Momyshuly Ave.), and T3 (Gogol St.–Bauyrzhan Momyshuly Ave.)—were strategically chosen to traverse key districts including Almaly, Auezov, and Bostandyk, in order to capture environmental conditions across both central urban zones and peripheral residential areas.

The design of the trolleybus routes deliberately incorporates endpoints located on opposite sides of the city, as it enables the assessment of air quality gradients from the densely populated core to the outlying neighborhoods. By measuring conditions at both the origin and terminus of each route, this study aimed to capture variations in occupancy, ventilation dynamics, and pollutant accumulation across the full transit journey.

This route layout provides broad geographic representation and allows for the analysis of how local microclimates, traffic conditions, and passenger turnover influence IAQ in semi-enclosed electric vehicles. Additionally, trolleybuses often operate with open windows and lack centralized HVAC systems, which makes them ideal testbeds for understanding natural ventilation effects and the impact of crowding during different times of the day.

**Figure 6:**
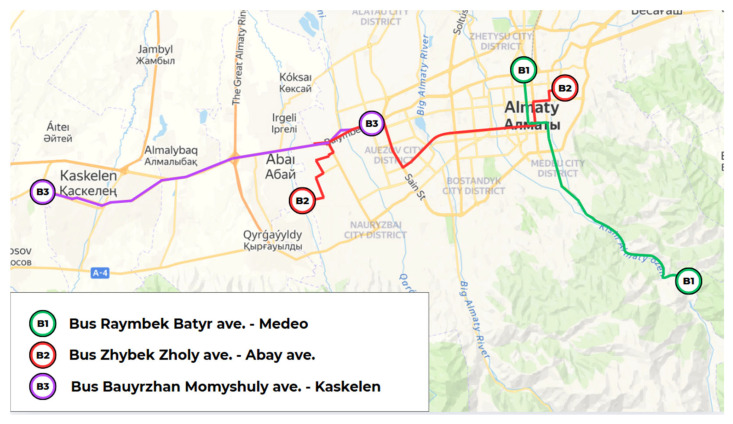
Bus routes B1–B3 cover urban-to-suburban and high-altitude corridors, which enables IAQ assessment across diverse microclimatic zones.

Figure 6 displays the bus routes selected for air quality monitoring, covering a diverse geographic span from the mountainous Medeu district in the east to the suburban town of Kaskelen in the west, as well as the southern urban periphery of Nauryzbay District. The three selected lines—B1 (Rayimbek Batyr Ave.–Medeu), B2 (Zhybek Zholy Ave.–Abay Ave.), and B3 (Bauyrzhan Momyshuly Ave.–Kaskelen)—were chosen to evaluate intra-urban and inter-urban variations in indoor air quality under real commuting conditions

Each route was selected based on its unique environmental and infrastructural context. Route B1 ascends into the elevated, forested region of Medeu, offering an opportunity to observe how altitude and topography influence pollutant levels in low-density, natural settings. In contrast, Route B3 connects Almaty to Kaskelen, a suburban town located beyond the city boundary, providing valuable data on long-distance commuter traffic and transitional microclimates between urban and rural zones. Finally, Route B2 traverses the southern districts of Almaty, specifically targeting Nauryzbay District, which is characterized by limited infrastructure and higher levels of residential sprawl, which makes it important for evaluating IAQ in underserved urban neighborhoods.

Collectively, these bus routes enable this study to capture longitudinal variation in pollution exposure, covering central business zones, mountain valleys, peri-urban expansion corridors, and poorly ventilated neighborhoods. This spatial diversity is essential for understanding how IAQ is modulated not only by occupancy and traffic flow but also by urban morphology and ventilation constraints across different parts of the city.

Together, the selected metro, trolleybus, and bus routes (Figure 4, Figure 5 and Figure 6) span a broad cross-section of Almaty’s urban fabric—from densely populated commercial centers and high-traffic transit hubs to peripheral districts with sparse infrastructure and even mountainous terrain. This multi-modal, multi-zonal coverage was deliberately designed to capture heterogeneous environmental exposures, assess spatial variability in indoor air quality, and ensure representativeness across infrastructural gradients. By incorporating both peak and off-peak sampling across these routes, this study provides a robust foundation for comparing pollution dynamics under realistic and demographically diverse commuting conditions.

### 4.2. Data Collection

Data collection was conducted in April 2025 in the form of two targeted one-hour sessions. These time windows were specifically chosen to capture maximally contrasting conditions—a peak load period (09:00–10:00) and an off-peak period (15:00–16:00)—in order to lay the foundation for research in this area and to test the effectiveness of the method. The first interval (08:00–09:00) corresponded to the morning rush hour, characterized by peak passenger density, while the second interval (10:00–11:00) represented off-peak conditions with reduced crowding. The use of one-hour windows aligns with prior studies on transport-related exposure, which suggest that short, consistent intervals are effective for capturing temporal variability while minimizing user and system burden [37,38]. For each transport mode—bus, trolleybus, and metro—separate sessions were conducted under similar external weather conditions to control for environmental variability.

Environmental data were collected using the Tynys IoT device, a custom-designed mobile platform for real-time IAQ monitoring in enclosed, mobile settings. The device was centrally mounted within the passenger area at a height of approximately 1.5 m, corresponding to the average breathing zone of a standing adult. Positioning the device at this height minimized local airflow artefacts and ensured readings mirrored typical human exposure.

**Figure 7:**
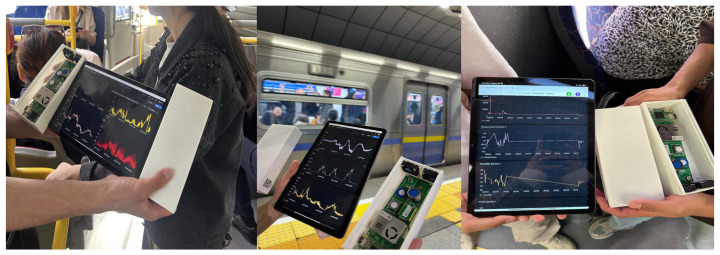
Deployment of the Tynys IoT device with real-time IAQ data visualization.

As shown in Figure 7, the Tynys IoT device was deployed during live monitoring sessions across buses, metro platforms, and metro cabins, with real-time IAQ metrics (PM_2.5_, PM_10_, CO_2_, temperature, humidity) being visualized on a connected tablet. In buses, the device was mounted on a central handrail away from windows and doors to avoid localized airflow, while, in trolleybuses, it was installed above the aisle between entry points to ensure symmetrical ventilation exposure; both vehicles operated with open windows and no air conditioning, relying on natural ventilation. In metro carriages, the device was placed on a central seat armrest within a fully enclosed environment with no natural airflow, where air recirculation depended solely on internal mechanical systems. These deployment settings enabled the system to capture pollutant dynamics under varying ventilation conditions, and the live visualizations in Figure 7 confirm the device’s robustness and stability during mobile, real-world data acquisition.

As illustrated in Figure 7, the Tynys IoT device was connected to a tablet displaying real-time graphs of indoor air quality (IAQ) metrics. On metro platforms, for example, particulate matter and gas concentration trends were visualized against the backdrop of arriving trains, which demonstrated the system’s capability for stable and clear data capture in dynamic environments.

During each one-hour session, the device continuously recorded five environmental parameters: carbon dioxide (CO_2_), particulate matter (PM_2.5_ and PM_10_), temperature and relative humidity (RH). All sensors operated autonomously at a fixed sampling interval, with data being stored locally for post-session analysis. In total, 18 measurement sessions were conducted—three routes and two time intervals per transport mode—yielding a robust dataset for both intra-modal and inter-modal comparisons.

No artificial interventions were introduced during fieldwork. All vehicles operated under standard service conditions, with natural passenger behavior, normal ventilation patterns, and typical stop frequencies. This design ensured that the recorded data accurately reflected real-world commuting environments, enhancing both the ecological validity and practical relevance of the findings.

### 4.3. Data Preprocessing

We performed a comprehensive preprocessing pipeline to standardize and validate the dataset before model training. The original dataset included multidimensional readings from environmental sensors collected at metro stations and platforms. The measured parameters comprised the carbon dioxide concentration (CO_2_), particulate matter (PM_2.5_ and PM_10_), temperature, and relative humidity (RH), as well as auxiliary metadata such as passenger flow and geographical location. All data were collected at fixed time intervals and annotated with the date and time, which enabled contextual analysis based on the time of day and station load.

To ensure numerical stability and comparability between features, the data were standardized using z-score normalization. Spatial categorical variables, such as location, were numerically encoded using label encoding to preserve the geographical context without compromising compatibility with machine learning algorithms.

As part of the classification task, each record was assigned to one of three air pollution categories: low, medium, or high. The thresholds for this categorization were defined based on guidelines from WHO, ASHRAE, and national air quality standards [1,4]. Pollution levels were classified as low when PM_2.5_ < 25 µg/m^3^ and PM_10_ < 50 µg/m^3^; as medium when PM_2.5_ ranged from 25 to 50 µg/m^3^ and PM_10_ from 50 to 100 µg/m^3^; and as high when either value exceeded these limits. This approach enabled consistent labeling of air quality conditions across different areas of public transport and provided a reliable foundation for training machine learning models.

To address the limited number of labeled samples and improve the robustness of the classification models, synthetic data generation was employed using the RegularSynthesizer model from the YData Synthetic SDK. Providing additional training instances allowed us to obtain synthetic data that helped bridge the gap caused by data scarcity, improving both the performance and resilience of the models. They are invaluable for data augmentation because they improve the generalization skills of models by exposing them to a wider range of scenarios [39]. RegularSynthesizer generates synthetic data by modeling the joint distribution of the original dataset’s features through decomposition into conditional probabilities, as defined in Equation (1):

(1) $$P\left(X\right)=\prod_{i=1}^{d}P\left(X_{i}\;\right|\;X_{<i}),$$

where X = (X1, X2, …, Xd) is the feature vector, and X < i denotes all features with indices less than iii. This approach preserves statistical dependencies between variables and enables realistic generation of new samples. The method is interpretable and efficient, and is especially suitable for small to medium-sized datasets. The original dataset contained 292 observations collected from indoor air monitoring in a metro environment. To enhance model training and ensure better generalization, the dataset was augmented to 10,000 samples.

In prior works, the inclusion of synthetic data alongside the original dataset led to a noticeable improvement in the accuracy of fault classification models. The augmented dataset enabled the models to generalize better to unseen fault scenarios, and thereby reduced the number of false positives and enhanced the detection precision [40]. Experimental results showed that the use of synthetic dataset led to substantial improvements in classification quality based on precision, recall, and F1-score metrics. This makes augmentation an optimal choice for enhancing reliability in situations with limited original data [41].

**Figure 8:**
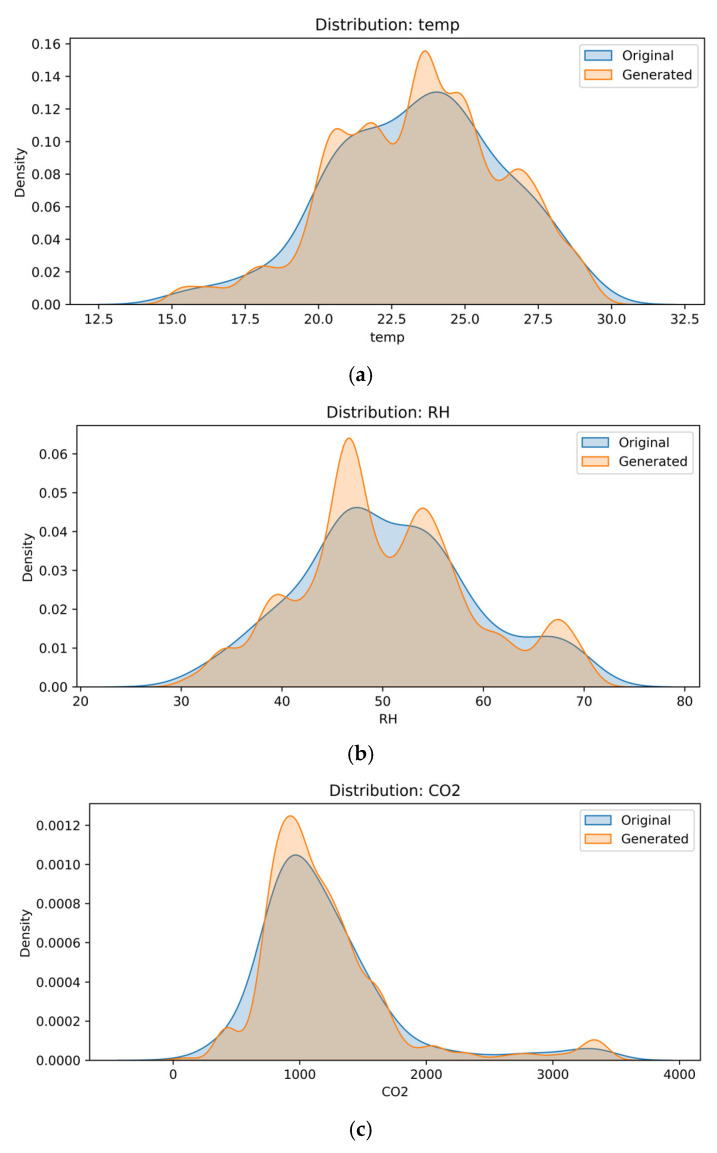

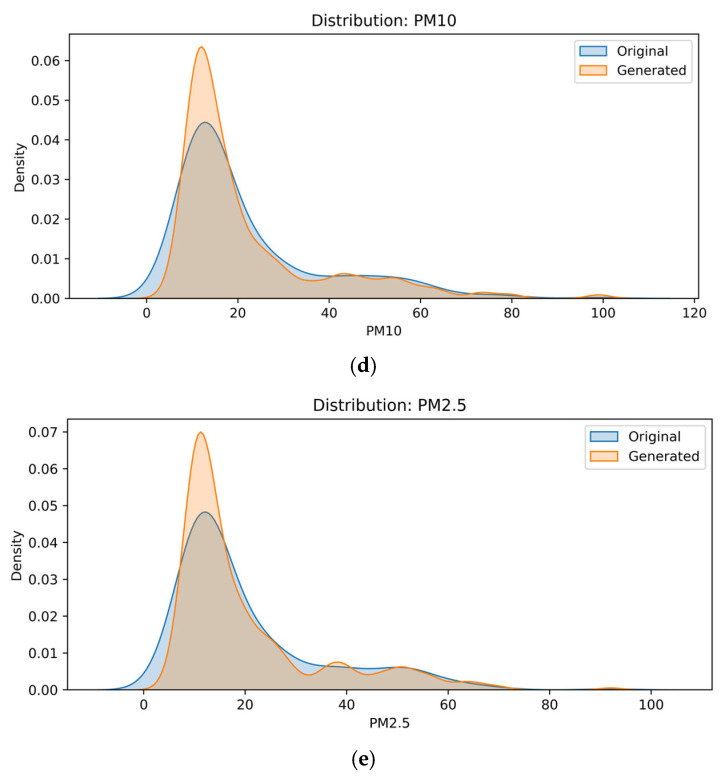
Distribution of environmental parameters: (**a**) temperature; (**b**) relative humidity; (**c**) CO_2_; (**d**) PM_10_; (**e**) PM_2.5_ in original vs. synthetic data.

The distribution of key features (CO_2_, PM_2.5_, RH, and temperature) in the original and synthetic datasets, shown in Figure 8, confirms that the generated samples closely follow the patterns and variability of the real-world data. The synthetic dataset preserved the multivariate relationships between environmental variables and enabled more balanced training of classification models, thereby improving the model’s generalization and helping to avoid overfitting.

The use of synthetic data significantly improved the class distribution, reduced the risk of model bias toward majority classes, and enhanced the generalization and robustness of the models under diverse real-world public transport conditions.

**Figure 9:**
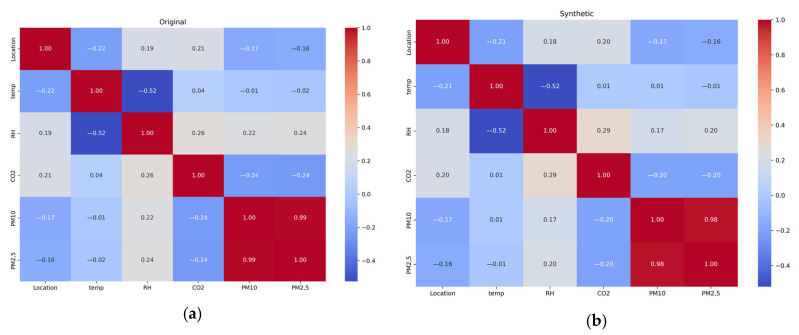
Correlation matrix between environmental parameters for: (**a**) original data, (**b**) synthetic data.

As shown in Figure 9, these correlation matrices vividly demonstrate the similarity in the relationships between parameters in the original and synthetic datasets. Analysis shows that the synthetic sample successfully reproduces the key correlational structures inherent in the source data, including a strong negative relationship between the temperature and relative humidity, as well as a high positive correlation between PM_10_ and PM_2.5_. Minor numerical discrepancies between the matrices indicate that the synthetic data retain the statistical properties of the original, while avoiding exact duplication.

To gain an initial understanding of the structure and characteristics of the dataset, an exploratory data analysis (EDA) was conducted on key environmental parameters collected from metro stations, buses, and trolleybuses. EDA is a preliminary analytical approach used to summarize the main characteristics of a dataset, often through visual methods and descriptive statistics. It helps reveal patterns, detect anomalies, test assumptions, and uncover relationships between variables before applying formal modeling techniques. In this case, the objective was to examine the distribution of features and identify potential correlations that may influence the accuracy of air pollution classification.

The dataset included continuous measurements such as CO_2_ concentration, temperature, and relative humidity (RH), as well as categorical variables including station location (Location), time interval (Time), and pollution level (Pollution Category). A statistical summary of the main features is presented in Table 1.

**Figure 10:**
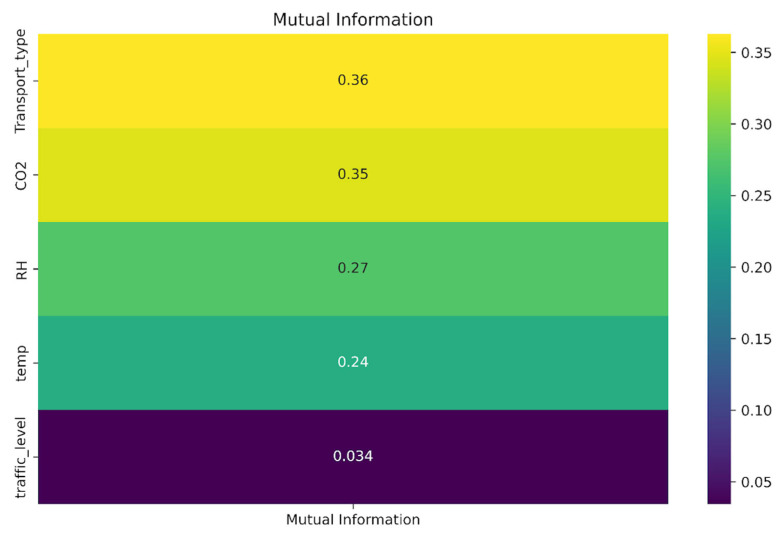
Pearson correlation matrix between environmental variables and pollution category.

To evaluate the relationships between features and the target variable, a mutual information (MI) analysis was conducted (Figure 10). The highest MI value was obtained for the variable Transport_Type (MI = 0.36), followed by CO_2_ (MI = 0.35), which indicates their high relevance for predicting pollution conditions. This may reflect the influence of the mode of transport on indoor air pollution levels.

Moderate MI values were also found for relative humidity (MI = 0.27) and temperature (MI = 0.24), which indicates that microclimatic conditions contribute to air quality formation. In contrast, the variable traffic level showed a minimal MI score (MI = 0.034), which suggests its limited impact on pollution levels in this context.

### 4.4. Machine Learning Classification

In this section of the research, supervised machine learning classification algorithms were employed to develop a model capable of assessing air pollution levels in public transport based on various environmental parameters. For this task, logistic regression (LR), random forest (RF), extreme gradient boosting (XGBoost), and decision tree (DT) models were selected due to their ability to handle complex data, non-linearity, and effectiveness in structured classification tasks.

Logistic regression [42] was used as a linear baseline method to identify simple first-order relationships between variables. Random forest [43] and decision tree [44], based on ensemble and rule-based tree structures, were applied to capture hierarchical patterns and non-linear feature interactions. XGBoost [45], a gradient-boosted tree ensemble, was selected for its high efficiency in handling structured tabular data and its ability to model complex higher-order feature interactions.

Preprocessing operations included the normalization of air quality parameters (CO_2_, PM_2.5_, and PM_10_) to ensure all variables were on the same scale. Additionally, the target variable representing air pollution levels was encoded into numerical values using LabelEncoder. The pollution levels were categorized as low, medium, or high, with label encoding applied: ‘0’ for low pollution, ‘1’ for medium pollution, and ‘2’ for high pollution. The dataset consisted of three features—CO_2_, PM_2.5_, and PM_10_—and a target variable representing the pollution level.

The classification models were developed using Scikit-learn for logistic regression, random forest, decision tree, and XGBoost for gradient boosting. To ensure the robustness and generalizability of the models, cross-validation with k-fold splitting (cv = 5) was performed, which allowed for systematic evaluation across multiple subsets of the data and the reduction of overfitting.

Additionally, to enhance the interpretability of tree-based models (random forest, decision tree, and XGBoost), SHAP (Shapley additive explanations) values were computed. SHAP is a machine learning interpretation method based on Shapley values from cooperative game theory. It is used to explain individual predictions by quantifying the contribution of each feature to the model’s output. SHAP helps determine both the magnitude and direction (positive or negative) of a feature’s impact on a specific prediction. This enables a deeper understanding of the model’s decision-making process and highlights the most influential factors that affect the outcome [46].

The performance of the machine learning models was evaluated using classification accuracy, precision, recall, and F1-score metrics to validate their effectiveness in assessing air pollution levels in public transport environments.

## 5. Results

To assess the impact of the passenger flow and time of day on the air pollution in public transport, we analyzed the pollutant levels in the subway, buses, and trolleybuses, focusing on key indicators such as the PM_2.5_, PM_10_, and CO_2_ concentrations. The highest air pollution was observed in the subway and buses, particularly during peak hours when the passenger density surged and ventilation systems struggled to maintain adequate air exchange. During these periods, the concentrations of PM_2.5_ and PM_10_ often exceeded 50 µg/m^3^ and 60 µg/m^3^, respectively, while the CO_2_ levels rose above 2800 ppm and approached 3000 ppm, which highlights the critical role of occupancy in driving pollutant accumulation. The limited effectiveness of ventilation systems in the subway and buses fails to cope with the sharp increase in pollutant concentrations in the cabin during high occupancy, which leads to the accumulation of harmful particles and gases in the air [47,48].

**Figure 11:**
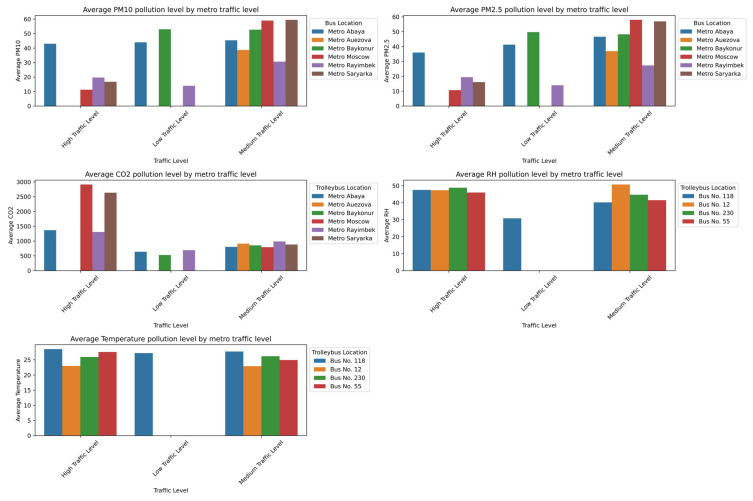
Comparison of PM_10_, PM_2.5_, CO_2_ pollution levels for metro and occupancy levels.

In the subway, the pollution peaks were most pronounced at densely trafficked stations such as Abay, Moskva, and Saryarqa, as shown in Figure 11. These stations, characterized by enclosed underground designs and limited mechanical ventilation systems [49], are structurally predisposed to the accumulation of pollutants. Consequently, the particulate matter (PM_10_ > 60 µg/m^3^; PM_2.5_ ≈ 50 µg/m^3^) and CO_2_ (>2800 ppm) concentrations reach their highest values during rush hours, posing potential health risks for passengers—especially those with respiratory or cardiovascular conditions. Although the relative humidity and temperature stay relatively stable, the humidity tends to decrease slightly under low-traffic conditions due to reduced human presence and evaporation. Overall, the unified analysis confirms that the peak occupancy, the time of day, and constrained ventilation collectively create critical conditions for elevated PM_2.5_, PM_10_, and CO_2_ levels in enclosed transport environments, which emphasizes the need for improved ventilation strategies and passenger flow management to mitigate health risks.

**Figure 12:**
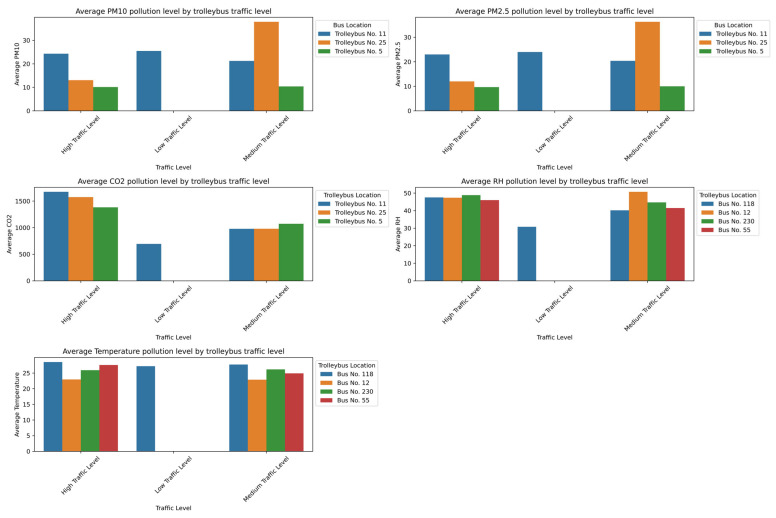
Comparison of PM_10_, PM_2.5_, CO_2_ pollution levels for trolleybus and occupancy levels.

The graphs in Figure 12 show that the air quality in trolleybuses is noticeably better, especially during low passenger flow in off-peak hours (10:00–11:00). Under low-traffic conditions, the levels of PM_2.5_, PM_10_, and CO_2_ are significantly lower—particularly in Trolleybus No. 5, where the particulate matter is near minimum levels and the CO_2_ drops to 600–700 ppm. This is due to a lower passenger density and more effective ventilation. Unlike the metro, trolleybuses likely have more open-air exchange systems, which prevent the buildup of pollutants. The humidity and temperature remain stable, which indicates that the passenger load is the primary factor that influences air pollution levels.

**Figure 13:**
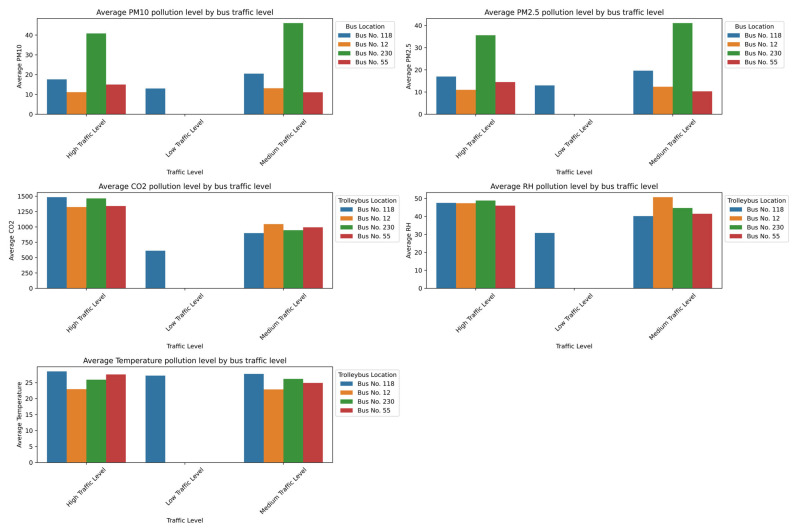
Comparison of PM_10_, PM_2.5_, CO_2_ pollution levels for bus and occupancy levels.

The graphs in Figure 13 show that, similar to the metro, the highest levels of air pollution in buses occur during peak hours. Under high-traffic conditions, the concentrations of PM_2.5_, PM_10_, and CO_2_ increase significantly. This is especially evident in Bus No. 230, where the particulate matter levels reach 40–50 µg/m^3^. The CO_2_ levels exceed 1300 ppm in nearly all buses during high occupancy, which indicates insufficient ventilation. The temperature and humidity remain relatively stable, although the humidity noticeably decreases under low-traffic conditions. Overall, the elevated pollution levels are closely linked to high passenger density and limited ventilation.

**Figure 14:**
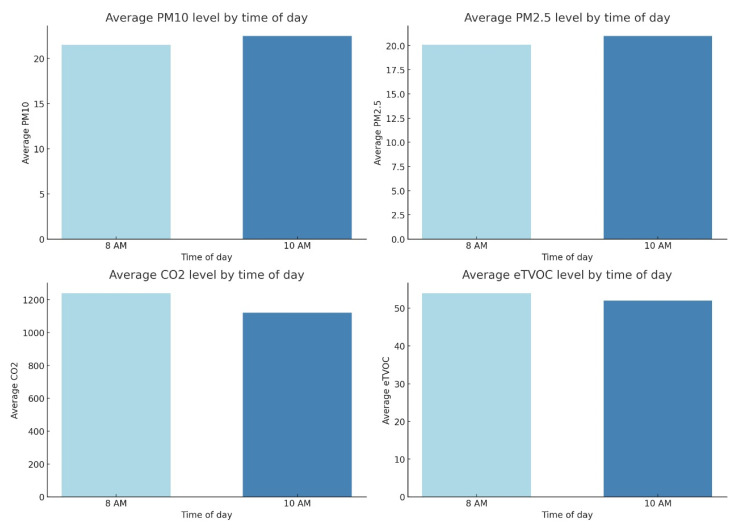
Average pollution levels by time of day.

Figure 14 presents a comparative study of the average values of key atmospheric parameters, including the concentrations of PM_10_, PM_2.5_, and CO_2_, the relative humidity (RH), and the temperature, recorded at two discrete time intervals: 8:00 and 10:00. Each graph visually demonstrates the juxtaposition of these parameters between the specified time points.

Data analysis revealed a systematic increase in the average concentrations of PM_10_, PM_2.5_, and CO_2_ at 10:00 compared to the measurements at 8:00. This trend can be interpreted as a consequence of the accumulation of pollutants in the near-surface atmospheric layer, potentially driven by increasing anthropogenic activity, including heightened traffic intensity, during the morning hours.

Concurrently, a decrease in the average relative humidity (RH) is observed from 8:00 to 10:00, which aligns with typical diurnal variations in meteorological parameters, where an increase in the air temperature leads to a reduction in the relative humidity, assuming constant absolute moisture content. Correspondingly, the average air temperature demonstrates a pronounced increase in the interval from 8:00 to 10:00, reflecting the natural process of daytime heating.

Collectively, the data presented in Figure 12 underscore significant diurnal fluctuations in atmospheric composition and microclimatic conditions. The identified increase in pollutant concentrations (PM_10_, PM_2.5_, CO_2_) at 10:00, alongside the concomitant changes in the RH and temperature, accentuates the dynamic nature of air quality and the necessity of considering temporal factors in its monitoring and management.

Following the analysis of air pollution levels, Table 2 provides a comprehensive summary of the performance of four different machine learning models: logistic regression, random forest, decision tree, and XGBoost. The table includes key metrics such as accuracy, precision, recall, and F1-score, all of which were calculated to evaluate the models’ effectiveness in accurately predicting air pollution levels based on the collected data. These metrics help assess how well each model performs in distinguishing between different pollution categories, such as low, medium, and high levels.

The random forest and XGBoost models demonstrated the best overall performance in terms of both their accuracy and F1-score. The XGBoost model achieved the highest accuracy at 0.9125 and an F1-score of 0.8054, which indicates its strong capability in capturing complex patterns within the data and effectively distinguishing between varying pollution levels. Its high precision (0.8295) and recall (0.7855) further reinforce its reliability in identifying both clean and heavily polluted air conditions, particularly in challenging environments such as enclosed public transport systems.

Closely following, the random forest model achieved an accuracy of 0.9082 and an F1-score of 0.8017, showcasing its robust performance and consistent ability to classify air quality levels. Its balanced precision (0.8198) and recall (0.7879) values highlight its effectiveness in maintaining low false-positive and false-negative rates, which makes it a dependable model for real-time air quality monitoring.

The decision tree model also performed competitively, with an accuracy of 0.9023 and an F1-score of 0.7694. While slightly lower than the ensemble methods, its results suggest a good trade-off between interpretability and predictive power, which makes it suitable for environments where model transparency is essential.

In contrast, the logistic regression model exhibited the weakest performance across all metrics, with an accuracy of 0.7814 and a notably lower F1-score of 0.4341. Its limited precision (0.4541) and recall (0.4346) suggest a reduced ability to distinguish between pollution levels, particularly under the complex, nonlinear conditions found in crowded or poorly ventilated transport environments. This outcome underscores the limitations of linear models when they are applied to real-world air quality data influenced by multiple interacting factors.

**Figure 15:**
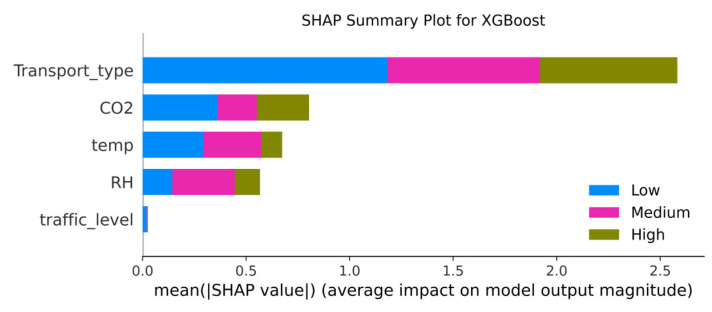
SHAP summary plot illustrating the average impact of features on the random forest model’s classification of pollution levels across classes (low, medium, high pollution).

To interpret the contribution of features to the classification process, the SHAP (Shapley additive explanations) model was applied. Figure 15 presents a summary plot showing the mean absolute impact of each feature on the output of the random forest model.

The presented SHAP diagram illustrates the contribution of key features to the air pollution level prediction using the XGBoost model. The most influential factor is *transport type, which highlights substantial differences in air quality across the metro, buses, and trolleybuses. This feature plays a particularly significant role in distinguishing between low, medium, and high pollution levels. The observed impact is likely related to differences in ventilation systems, passenger density, enclosure design, and vehicle architecture.

The second most important feature is CO_2_ concentration, which consistently contributes to predictions across all pollution classes. This confirms its role as a reliable indicator of air quality, particularly in enclosed environments with high passenger density.

The temperature and relative humidity also contribute to the model’s predictions, especially for higher pollution levels, although their influence is less pronounced. The traffic level feature shows the least impact, which suggests that it plays a secondary role in this dataset compared to environmental and structural factors.

In summary, the analysis confirms that the transport type and CO_2_ concentration are the primary predictors of air pollution levels in public transport, while the effects of the temperature, humidity, and traffic load are more limited and context-dependent.

**Figure 16:**
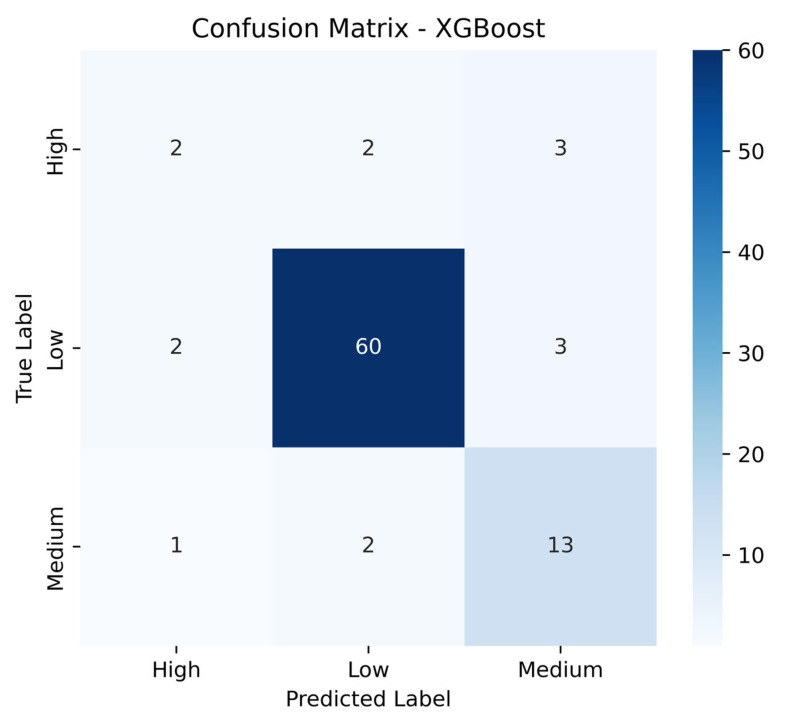
Confusion matrix for XGBoost model.

Figure 16 presents the confusion matrix for the XGBoost model, which was used to classify air pollution levels into three categories: low, medium, and high. The matrix provides a detailed overview of the model’s classification performance and highlights areas where improvements are needed.

The model shows high accuracy in identifying low pollution levels, correctly classifying 1879 instances, which confirms its robustness in detecting clean air conditions—typically associated with well-ventilated or less crowded transport such as trolleybuses. However, its performance declines when predicting medium pollution levels, where only 323 cases were correctly identified, while 82 were misclassified as low and 34 as high. This indicates that medium-level pollution shares overlapping features with the other two classes, which complicates the classification process.

For the high pollution category, the model achieves 123 correct predictions, but 65 instances were confused with medium and 4 with low levels, revealing moderate performance in this class.

Despite these misclassifications, XGBoost demonstrates strong overall performance, particularly in detecting low pollution, which aligns with previous evaluation metrics such as the accuracy and F1-score (as presented in Table 2). These results further support the model’s suitability for deployment in complex transport environments—such as metros and buses—where pollution levels are dynamic and influenced by factors like the ventilation quality and passenger load.

## 6. Discussion

The United Nations Development Programme (UNDP) in Almaty, through the ‘Green and Safe Streets’ initiative, emphasized the need for comprehensive measures to address air pollution from the city’s transport sector. As public transport remains a significant contributor to harmful emissions, the initiative highlighted the importance of upgrading ventilation systems in older vehicles [50]. Previous research focusing on the indoor environmental quality (IEQ) in passenger transport vehicles across tropical and subtropical regions identified poor indoor air quality (IAQ) as a significant health concern. Common contributing factors included overcrowding, insufficient ventilation, and exposure to airborne pollutants such as PM_2.5_ and volatile organic compounds (VOCs) [1]. In our study, the highest levels of pollution occur during peak hours, when passenger density is at its highest. During these times, there is a significant rise in the concentration of pollutants such as CO_2_, PM_2.5_, and PM_10_. During off-peak hours, the pollution levels decrease as the passenger flow significantly is significantly reduced, which allows ventilation systems to work more efficiently and the air in the cabin to be cleaned more quickly [17,18]. With fewer passengers, the air has more space to circulate and disperse pollutants, which leads to a noticeable improvement in the overall air quality. Our research indicated that CO_2_ was strongly correlated with occupancy and it was proven to be the most informative parameter. The PM_10_ concentration, however, often correlated with the dynamics of people when getting on and off, but not with the occupancy [51]. Because these corridors consistently operate at or above capacity, mitigating crowd-related pollution peaks is a high-priority public-health issue for Almaty residents.

To improve the air quality of public transport, it is necessary to focus on enhancing air circulation systems and implementing real-time air quality monitoring systems to maintain a healthy environment for passengers. In areas with high pollution, it is important to install more effective filtration and ventilation systems, which will help reduce the accumulation of pollutants and gases. As part of this management, the passenger flow should be optimized, and overcrowding in vehicles should be reduced, particularly during peak hours, through strategic scheduling and the use of larger or more frequent vehicles. To improve the air quality in public transport, it is recommended to invest in enhanced ventilation systems that will help reduce the amount of particles and harmful gases. The implementation of real-time air quality monitoring systems will enable a prompt response to high pollution levels and inform both passengers and operators about the deterioration in air quality. Such targeted measures on Almaty’s busiest routes could yield immediate benefits and serve as a scalable model for similarly crowded corridors elsewhere in the city.

Such targeted interventions are only effective when supported by consistent, real-time environmental data to inform decision-making and evaluate outcomes. In this context, the deployment of low-cost, portable air quality monitoring devices becomes essential for tracking pollutant levels across dynamic public transport environments.

While the validation results confirmed close agreement between the Tynys device and the Qingping reference monitor over a 24-h field session, several sensor-level considerations merit discussion. The MH-Z19C NDIR sensor and ZH06 laser particle counter are widely used in mobile environmental monitoring due to their low cost and reasonable data consistency. However, prior studies have reported that their readings can be influenced by environmental factors such as the temperature and humidity, especially under the dynamic conditions typical of mobile deployment [52,53]. In the current study, this potential cross-sensitivity was partially mitigated by integrating the ZS05 temperature and humidity probe, which enabled post-hoc correlation analysis and future compensation strategies.

Regarding inter-device correlation, the MH-Z19C sensor’s ±50 ppm specification has been validated under stable laboratory settings in previous comparative studies against reference analyzers [52]. While our calibration session was limited to one hour, the device demonstrated strong agreement in terms of its trend and magnitude with the Qingping Air Quality Monitor Gen 2, which itself conforms to industrial-grade IAQ monitoring standards. Although long-term drift was not explicitly assessed in this study, the short-term consistency across key parameters—CO_2_, PM_2.5_, PM_10_, temperature, and RH—provides confidence in the Tynys device’s suitability for real-time, operational monitoring.

While the validation of the Tynys device was performed via co-location with a commercial sensor rather than a certified reference instrument, the resulting data are nonetheless valuable within the context of this pilot study. The primary objective was to assess the feasibility of and establish a proof-of-concept for using low-cost sensors for air quality monitoring, not to establish metrological traceability. The strong inter-device correlation and consistent tracking of environmental fluctuations demonstrate that these sensors are suitable for detecting relative changes and temporal trends in air quality. This capability is fundamental for applications such as identifying pollution hotspots, understanding the impact of factors like passenger density, and triggering real-time alerts. While these sensors may not provide the absolute accuracy of a reference instrument, their validated ability to track fluctuations confirms their potential for deployment in scalable, wide-area monitoring networks where the emphasis is on relative air quality assessment and pattern recognition rather than metrological precision. These findings provide a valuable basis for continued system enhancement and broader application, especially in the Central Asian region, where elevated pollution levels constitute a well-documented and ongoing threat to public health, as established in prior studies.

There are some limitations to the study that should be acknowledged. The data were collected exclusively in Almaty (Kazakhstan) across nine predetermined public transport routes, with the measurements being confined to two one-hour windows during a single month (April 2025). This design excludes broader spatial coverage and omits seasonal and diurnal variability, which are known to significantly affect air pollution levels. Additionally, key environmental and operational factors such as the vehicle age, ventilation settings, and external meteorological conditions were not controlled, which makes it difficult to isolate the effect of the passenger density on the indoor air quality. Although the Tynys IoT device was validated against a reference-grade monitor, the validation was short-term and limited in its pollutant range, which leaves questions regarding sensor drift and long-term data consistency unresolved. The dataset used for analysis contained only 292 real observations and was synthetically expanded to 10,000 records using the RegularSynthesizer, which potentially introduced artefacts and biased the model’s performance. This risk is compounded by the black-box nature of generative models, which often lack transparency and are difficult to interpret. Interpretability remains a challenge, even with explainable AI tools like SHAP, which may not fully capture how synthetic data are generated. Standard auditing methods are often insufficient to assess the realism and reliability of data, and there is a lack of domain-specific benchmarks for evaluation [54].

## 7. Conclusions

The findings from this pilot study offer a preliminary assessment of the air quality within the enclosed spaces of Almaty’s public transport. Our measurements indicate that the pollutant concentrations can reach high levels during peak hours. Specifically, the PM_2.5_ in bus cabins and metro cars reached levels as high as 50 µg/m^3^. Concurrently, we found that the CO_2_ levels in metro cars routinely exceeded 1500 ppm during these periods, which suggests rapid accumulation in the confined underground environment. By contrast, during off-peak hours, the CO_2_ and PM_2.5_ concentrations inside trolleybus cabins were substantially lower than those observed in the metro and buses. This likely reflects the benefits of lower passenger density and more effective ventilation. These findings represent a preliminary step toward the real-time monitoring of peak and off-peak loads for long-term studies in this area. Further, more extensive research is needed to establish a complete picture of year-round dynamics and to formulate comprehensive recommendations. To address these challenges, this study introduced a real-time monitoring system that integrates a custom-designed, low-cost IoT device (Tynys) with machine learning algorithms. The Tynys device captures minute-by-minute measurements of CO_2_, PM_2.5_, and PM_10_ levels, the temperature, and the relative humidity, enabling the high-resolution spatiotemporal analysis of pollution dynamics in public transport vehicles. This granular data collection is especially valuable for rapidly growing urban centers like Almaty, where real-time environmental surveillance remains underdeveloped.

The XGBoost model achieved the best performance, attaining 91.25% accuracy in classifying pollution levels across the three transport modes, which makes it highly effective for real-time air-quality monitoring. The model reliably distinguished between high, medium, and low pollution conditions, a capability that is critical for implementing timely measures to improve air quality. This strong accuracy demonstrates that XGBoost can be deployed for operational monitoring within public transport systems. Crucially, the proposed framework extends beyond passive monitoring. It establishes the basis for an intelligent and automated control system in which the machine learning model functions as an adaptive decision-making core. It will not only function as an intelligent control platform for drivers and system operators but also offer a passenger-facing component. By displaying real-time information on air quality and occupancy levels across various transport modes, the system will enable commuters to make informed decisions regarding which vehicles to board. Passengers will be able to identify cleaner and less crowded options, which will thereby reducing individual exposure risks and promote a more balanced distribution of occupancy across the fleet. This added transparency and agency will contribute to improved public health outcomes, enhance user trust, and support the broader adoption of intelligent transport infrastructure. Such functionality is particularly critical in cities like Almaty, where aging transit systems and elevated pollution levels create unique health and mobility challenges for the urban population. Nonetheless, the system’s implementation presents several technical and operational challenges. These include initial capital costs for retrofitting older vehicles, the need for ongoing sensor maintenance and calibration, wireless connectivity issues in underground settings, and cybersecurity risks. Additionally, model drift over time due to evolving environmental patterns and passenger behaviors necessitates periodic retraining to maintain predictive accuracy.

The limited duration of the data collection campaign, restricted to two one-hour intervals in April 2025, constrains the temporal generalizability of the findings. While these windows captured clear contrasts between peak and off-peak conditions, they do not account for seasonal variation, weekday–weekend dynamics, or weather-related changes in air quality and ventilation behavior. Consequently, the current results should be interpreted as a representative case study rather than a comprehensive characterization of year-round exposure.

The strong, promising results do not eliminate this study’s methodological limitations. Future work will extend monitoring across multiple cities and seasons, capture detailed operational factors, carry out long-term calibration of the Tynys sensors, and build analyses on larger, organically collected datasets instead of synthetically augmented ones. It will also prioritize a comprehensive longitudinal validation study to assess the long-term performance and reliability of the Tynys device under real-world operational conditions. This extended evaluation will enable the characterization of sensor drift, sensitivity to cumulative exposure to vehicle exhaust, and degradation due to persistent mechanical stressors such as the vibration and temperature fluctuations typical of mobile environments. By capturing data over several weeks or months, this study will provide critical insights into the durability and stability of the sensing components, thereby ensuring the robustness of the Tynys platform for continuous deployment in public transport systems and reinforcing its value for urban mobility and public health monitoring. Moreover, future research should emphasize the importance of proper ventilation for both public health and energy efficiency. To support this, we plan to integrate technologies such as LoRaWAN or NB-IoT for the efficient, real-time monitoring and control of ventilation systems. In addition, we aim to explore advanced deep learning methods to enhance the system’s predictive capabilities and enable more robust, context-aware decision-making across diverse environmental and operational conditions.

## Figures and Tables

**Table 1:** Summary statistics of key environmental features collected from public transport.

Feature	Mean	Std. Dev.	Min	25%	Median	75%	Max
Temperature (°C)	23.37	2.89	15.30	21.38	23.60	25.23	29.40
Relative Humidity (%)	50.58	8.67	30.80	45.38	49.90	55.80	70.30
CO_2_ (ppm)	1194.93	556.40	96.00	865.75	1059.00	1361.25	3410.00
PM_10_ (µg/m^3^)	21.82	16.22	5.00	11.00	15.00	26.00	99.00
PM_2.5_ (µg/m^3^)	20.43	14.76	5.00	11.00	14.00	25.00	92.00

**Table 2:** Performance of each model in predicting air pollution in public transport.

Title 1	Accuracy	Precision	Recall	F1-Score
Logistic Regression	0.781397	0.454087	0.434599	0.434076
Random Forest	0.908163	0.819759	0.787901	0.801668
Decision Tree	0.902276	0.793215	0.749375	0.769371
XGBoost	0.912480	0.829526	0.785522	0.805447

## Data Availability

Data are contained within the article.

## Abbreviations

The following abbreviations are used in this manuscript:

IAQ	Indoor Air Quality
IoT	Internet of Things
HVAC	Heating, Ventilation, and Air Conditioning
PM_2.5_	Particulate Matter with diameter ≤ 2.5 µm
PM_10_	Particulate Matter with diameter ≤ 10 µm
CO_2_	Carbon Dioxide
VOCs	Volatile Organic Compounds
ML	Machine Learning
XGBoost	Extreme Gradient Boosting
MQTT	Message Queuing Telemetry Transport
SQL	Structured Query Language
CFD	Computational Fluid Dynamics
SCADA	Supervisory Control and Data Acquisition
WHO	World Health Organization
RH	Relative Humidity
T1–T3	Trolleybus Routes 1–3
B1–B3	Bus Routes 1–3
M1–M3	Metro Segments 1–3
